# Draft Genome Sequence of Isolate POC01, a Novel Anaerobic Member of the *Oscillospiraceae* Family, Isolated from Human Feces

**DOI:** 10.1128/mra.01134-21

**Published:** 2022-01-20

**Authors:** Victoria Meslier, Florian Plaza Oñate, Maryne Ania, Mélanie Nehlich, Ilia Belotserkovsky, Samuel Bellais, Vincent Thomas

**Affiliations:** a Bioaster, Institut de Recherche Technologique, Paris, France; b Université Paris-Saclay, INRAE, MGP, Jouy-en-Josas, France; University of Maryland School of Medicine

## Abstract

We report the isolation, culture, and genome sequencing of isolate POC01, a strictly anaerobic bacterium isolated from a healthy donor, representing a previously uncultured member of the *Oscillospiraceae* family.

## ANNOUNCEMENT

*Oscillospiraceae* bacteria are prevalent members of the human gut microbiota that have been regularly associated with health markers, such as microbial richness and leanness, and are thought to present anti-inflammatory effects (1–3). Here, we report the draft genome sequence of isolate POC01, a strictly anaerobic bacterium that was isolated from a healthy donor from whom consent was obtained.

A fecal specimen was collected into a sterile pouch containing an AnaeroGen bag (Oxoid) and was processed within 2 h. In an anaerobic chamber filled with 90% N_2_/5% CO_2_/5% H_2_ (Bactron 600 chamber; Sheldon), 1g of sample was fully homogenized in reduced phosphate-buffered saline (PBS) (10 mL) using 2.4-mm glass beads and a vortex mixer. The suspension was diluted 10-fold, filtered through a 70-μm cell strainer, washed once with PBS, diluted 100-fold in PBS, and stained using Syto-62 DNA dye and vancomycin-BODIPY-FL as a Gram-positive dye, as described previously (4). Anaerobic sorting of small Gram-positive cells was performed using the BD Influx cell sorter (4) on reduced modified Gifu anaerobic medium (mGAM) CRIM plates (HyServe) supplemented with 1 g/L cellobiose, 1 g/L inulin, 2.5 g/L mucin, and 30% bovine rumen. After 3 days of incubation at 37°C under strict anaerobic conditions, isolate POC01 was grown in mGAM CRIM broth for 48 h in the anaerobic chamber and subjected to DNA extraction.

**FIG 1:**
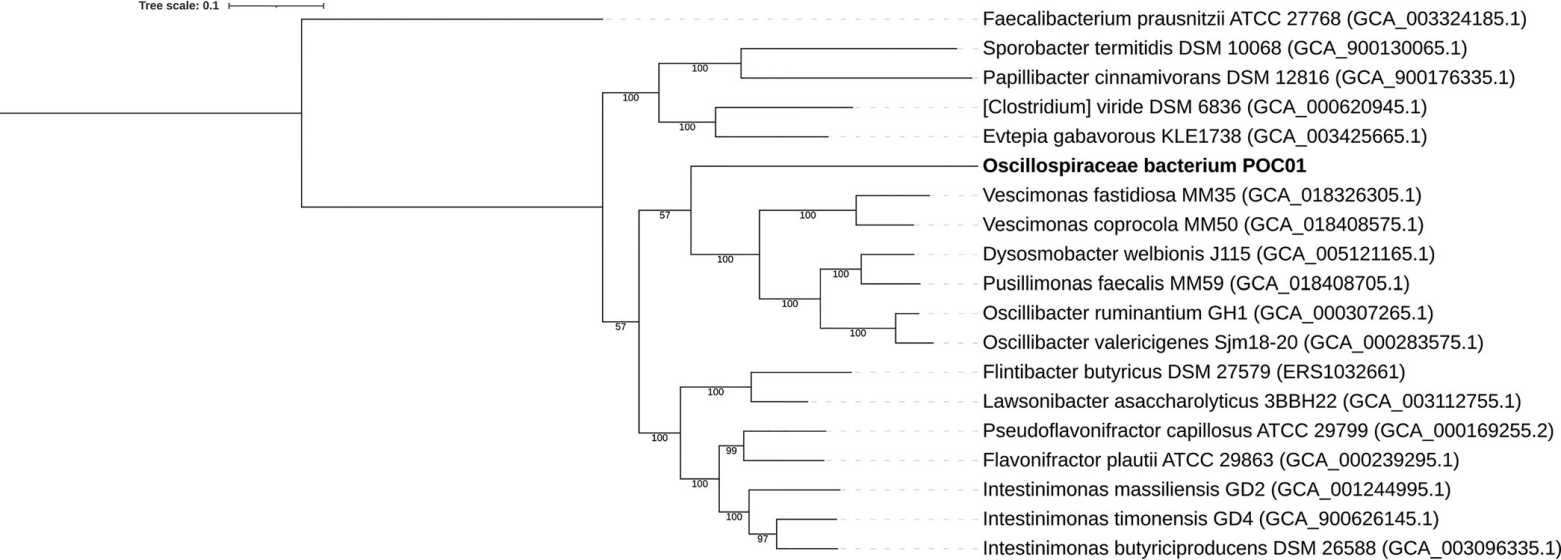
Phylogenetic tree obtained from multilocus sequence analysis (MLSA). GenBank accession numbers are indicated in parentheses for assemblies derived from type strains. Bootstrap support values are reported for each branch.

Genomic DNA was extracted with the DNA fungal and bacterial miniprep kit (Zymo Research, USA) following the manufacturer's guidelines. The sequencing library was generated using the NEBNext Ultra DNA library preparation kit for Illumina (New England Biolabs, USA) following the manufacturer’s recommendations. Genome sequencing was performed on an Illumina NovaSeq 6000 device, which produced 16,167,159 paired-end reads of 150 bp. Below, default parameters were used for all software unless otherwise specified. Sequencing data quality control was performed with fastp v0.23.0 (5), and high-quality reads were subsequently assembled using SPAdes v3.15.3 (6) (--isolate option). Contigs shorter than 1,000 bp or with coverage of <5× were filtered out. Final assembly statistics computed with CheckM v1.1.3 (7) are given in Table 1. A full-length 16S rRNA gene (1,519 bp) was extracted from the genome with RNAmmer v1.2 (8) and aligned against the SILVA v138 database (9) with BLASTn v2.10.1 (10). The best hit (identity, 99.7%; coverage, 98%) corresponded to a gene (GenBank accession number DQ904727.1) assigned to the *Ruminococcaceae* UCG-005 group. Consistently, taxonomic annotation with GTDB-Tk v1.5.0 (11) assigned the genome to an uncharacterized species named *Oscillospiraceae* CAG-170 sp900549635, for which no genome sequences derived from isolates are available. Multilocus phylogenetic analysis was performed with 39 universal marker proteins extracted from the POC01 genome and closely related strains using fetchMGs v1.2 (12). Multiple sequence alignment was achieved with MUSCLE v3.8.31 (13) and curated with trimAl v1.4.rev22 (14) (–automated1 mode). One thousand bootstrap alignments were generated with Goalign v0.3.5 (15). Phylogenetic trees were generated with FastTree v2.1.10 (16) (–gamma –slownni) and visualized with iTOL (17) (Fig. 1). Finally, assembled sequences were annotated with the PATRIC Genome Annotation Service (18).

**TABLE 1:** Final assembly statistics

Parameter	Value
Total length (bp)	3,213,277
No. of scaffolds	78
GC content (%)	55.3
Mean coverage (×)	1,205
Size of longest scaffold (bp)	249,011
*N*_50_ (bp)	93,582
*L*_50_	11
No. of coding sequences	3,420
No. of RNAs	52
Estimated completeness (%)	94.63
Estimated contamination (%)	1.48

### Data availability.

Sequencing data were deposited in the European Nucleotide Archive (ENA) under BioProject accession number PRJEB47943. The genome assembly is available in GenBank under accession number GCA_916988195.
